# Care burden and associated factors in caregivers of children with cancer

**DOI:** 10.1186/s13052-022-01291-w

**Published:** 2022-06-13

**Authors:** Mahnaz Chaghazardi, Maryam Janatolmakan, Shahab Rezaeian, Alireza Khatony

**Affiliations:** 1 Student Research Committee, Kermanshah University of Medical Sciences, Kermanshah, Iran; 2 Social Development and Health Promotion Research Center, Health Institute, Kermanshah University of Medical Sciences, Kermanshah, Iran; 3 School of Health, Kermanshah University of Medical Sciences, Kermanshah, Iran; 4 Infectious Diseases Research Center, Kermanshah University of Medical Sciences, Kermanshah, Iran

**Keywords:** Caregivers, Child, Caregiver burden, Cancer

## Abstract

**Background:**

Evidence suggests that many parents who care for their children with cancer are affected by the care burden due to the chronic nature of the disease. The aim of this study was to determine the burden of care level and its related factors in the caregivers of children with cancer.

**Method:**

A total of 270 caregivers of children with cancer were included in this cross-sectional study by convenience sampling method. Data collection tools were a personal information form and the Novak & Guest’s Caregiver Burden Inventory. Data were analyzed by descriptive and inferential statistics (chi-square and univariate linear regression model).

**Results:**

The mean ages of caregivers and patients were 35.7 ± 7.0 and 3.1 ± 1.6 years, respectively. The mean care burden was 68.4 ± 1.5 out of 120. About 50 and 36% of caregivers had moderate and severe care burden, respectively.

**Conclusion:**

Caregivers had moderate to high care burden. A number of factors associated with care burden were identified. Health authorities need to take family-centered measures to reduce the burden of caregivers.

## Introduction

In recent decades, the prevalence of cancer in children has increased [1] so that more than 160,000 children are diagnosed with cancer every year [2]. According to the World Health Organization, the annual incidence of cancer in children is 100 per million [3]. Cancer is responsible for the death of 13% of children aged 5 to 15 in Iran [4]. Today, with the advancement of medical sciences, the survival rate of children with cancer has increased [5], and this issue has affected various aspects of life in the caregivers of these children [6]. In Asian societies, the caregivers of children with cancer are usually family members who are present at all stages of the diagnosis and treatment of the disease [6, 7]. Many parents who care for their children with cancer are affected by the care burden due to the chronic nature of the disease [8, 9].

Care burden is the physical, mental, social, or financial response that occurs to meet these demands due to an imbalance between care demands and resources of the caregiver [10, 11]. Care burden can affect the physical, mental, spiritual, and social health of the caregiver [12]. In this regard, the results of a study (2017) in Iran showed that the caregivers of cancer patients face several challenges such as ignorance, instability, anxiety, helplessness, confusion, and stress [13]. High care burden may reduce the provision of care to patients, and therefore exacerbate their condition [7, 10, 13]. Exacerbation of patients’ clinical status can also lead to increased care burden. This vicious cycle causes many physical and psychological consequences for caregivers [7]. Evidence suggests that caregivers of children with cancer experience high levels of care burden [4, 8, 9, 13–18].

Numerous sociodemographic factors affect the amount of care burden perceived by caregivers, including age, gender, marital status, patient’s age, education, occupation, and monthly income. Regarding the factors related to caring burden in the caregivers, studies have shown different results [4, 8, 9, 14–16, 18–20]. Since the first step in helping the caregivers with care burden is to identify them [7, 10, 11], identifying caregivers with care burden will play a crucial role in promoting their health and improving the quality of care provided to the patient [21]. Due to the limited number of studies that have examined the care burden and related factors in the caregivers of children with cancer in Iran, the current study was performed to determine the level of care burden and the factors associated with it in the caregivers of children with cancer.

## Material and methods

### Study design

The present study is a cross-sectional descriptive-analytical study. In cross-sectional studies, it is not possible to determine the cause-and-effect relationships between the study variables because the exposure and outcome variables are collected simultaneously [22]. The study was designed and reported based on the Strengthening the Reporting of Observational Studies in Epidemiology (STROBE) guidelines [23].

### Sample and sampling method

The study population included the caregivers of children with cancer admitted to the oncology ward of Mohammad Kermanshahi Hospital in Kermanshah, western Iran. This hospital with 70 active beds is the main center of pediatric cancer in western Iran. Based on the results of the study of Lee et al. (2015) [24], with 95% confidence and 80% power, 245 people were estimated using the correlation coefficient formula, and with a non-response probability, 10% was added to the final sample size. Finally, 270 participants were included in the study by convenience sampling method. The inclusion criteria were having a child with cancer, being literate, and consent to participate in the study.

### Instruments

The Study instruments included a personal information form and the Novak & Guest’s Caring Burden Inventory (CBI). The personal information form consisted of two parts. The first part included four questions on the characteristics of hospitalized children in terms of age, sex, type of cancer, and duration of cancer. The second part, with eight questions, was dedicated to the characteristics of the caregivers and included questions about age, sex, marital status, education, occupation, monthly income, family relationship with the patient, and average time of patient care. CBI was developed by Novak & Guest (1989) [25]. This tool has been psychometrically assessed in previous studies. Shin et al. (2017) assessed the internal consistency of the questionnaire and reported a Cronbach’s alpha level of 89.0 [26]. The Persian version of this instrument has been psychometrically evaluated in Iran, with a Cronbach’s alpha coefficient of 0.92 [27].

The CBI has 24 items and consists of five subscales, including time-dependent (questions 1 to 5), evolutionary (questions 6 to 10), physical (questions 11 to 14), social (questions 15 to 19), and emotional (questions 20 to 24). The items are of the five-point Likert type, including completely false, false, somewhat true, true, and absolutely true, which are rated from one to five, respectively. Examples of questionnaire items are “I do not get enough sleep”, “my health is affected”, and “I feel physically tired”. The score range of the questionnaire is from 24 to 120. The individuals are classified into mild (24–47), moderate (48–71), severe (72–95), and very severe (96–120) categories [25, 27].

### Data collection

To collect data, the researcher referred to the oncology department of Mohammad Kermanshahi Hospital every day and included the qualified caregivers in the study. For this purpose, the objectives of the study were first stated to the caregivers, and if they were satisfied, the questionnaires were provided to them and collected after completion.

### Statistical analysis

Data were analyzed by SPSS-18 software using descriptive and inferential statistics. In the descriptive part, mean, standard deviation and frequency distribution were used to describe the variables. Chi-square test and linear regression model were used in the inferential part. The chi-square test was used to determine the relationship between sociodemographic variables in terms of the categories of care burden intensity. Univariate linear regression model was used to determine the factors related to care stress (as a quantitative variable). The desired level of significance was < 0.05.

### Ethical considerations

The ethics committee of Kermanshah University of Medical Sciences approved the study with the code IR.KUMS.REC.1397.1016. The objectives of the study were explained to all participants, and informed written consent was obtained from them. The participants were reassured about the confidentiality of their personal information.

## Results

The mean age of patients was 3.1 ± 1.6 years. Most patients were male (*n* = 142, 52.6%), and the most common type of cancer was acute lymphoblastic leukemia (*n* = 193, 71.5%). The mean duration of cancer diagnosis was 0.6 ± 0.7 months. The mean age of caregivers was 35.7 ± 7.0 years, and most of them were female (*n* = 189, 70%), married (*n* = 244, 90.4%), and parents of children (*n* = 246, 91.2%) and had high school diploma (*n* = 98, 36.3%) and insufficient income (*n* = 257, 95.2%). The maximum amount of care was 19–24 hours (*n* = 133, 14.3%) (Table 1).

**Table 1:** Demographic characteristics of caregivers (*N* = 270)

Variables	Subgroup	n (%)
Age (year)	< 40	216 (80.0)
	≥40	54 (20.0)
Gender	Male	81(30.0)
	Female	189(70.0)
Marital Status	Single	26(9.6)
	Married	244(90.4)
Relationship with patient	Parent	246(91.2)
	Sibling	12(4.4)
	Others	12 (4.4)
Education	Primary	77(28.5)
	Secondary	53(19.6)
	High school diploma	98(36.3)
	Academic	42(15.6)
Job	Unemployed	195 (72.2)
	Employed	22 (8.2)
	Self-employed	53 (19.6)
Income	Insufficient for expenses	257(95.2)
	Sufficient for expenses	13(4.8)
Average caring time (hours)	1–6	30 (11.1)
	7–12	64 (23.7)
	13–18	43 (15.9)
	19–24	133 (49.3)

The mean care burden in the caregivers was 68.4 ± 1.5 out of 120. About 51.0% (*n* = 140) and 36.0% (*n* = 97) of caregivers had moderate and severe care burden, respectively (Table 2). The results of univariate linear regression model showed that the care burden was 8.5 times higher in the female caregivers than in male caregivers on average (*p* = .004). The single caregivers had significantly higher care burden than the married ones (*p* = .003). The level of care burden in the caregivers who were second-degree relatives of the patient was significantly lower than those who were the first-degree relatives of the patient (*p* = .005). The results showed that care burden decreased with a rise in education level (*p* = .001). The employed caregivers had less care burden than unemployed caregivers (*p* = .031). Further, as the number of hours of patient care increased, the amount of care burden increased significantly (*p* < .001). Moreover, the amount of care burden decreased by an average of 0.9 units with one year increase to the patient’s age, which was statistically significant (*p* < .001). Patient’s gender and caregiver’s income level did not predict care burden (Table 3).

**Table 2:** Frequency distribution of care burden in terms of sociodemographic variables of caregivers (*N* = 270)

Variables	Subgroup	Intensity of care burden				*p*-value
		Low, n (%)	Middle, n (%)	High, n (%)	Very high, n (%)	
Age	< 40	15 (6.9)	108 (50.0)	82 (37.9)	11 (5.2)	.267
	≥40	6 (11.2)	32 (59.2)	15 (27.8)	1 (1.8)	
Gender	Male	9 (11.0)	48 (59.3)	22 (27.2)	2 (2.5)	.095
	Female	12 (6.3)	92 (48.7)	75 (39.7)	10 (5.3)	
Relationship with patient	Parent	15 (6.1)	126 (51.3)	94 (38.2)	11 (4.4)	.016
	Sibling	3 (25.0)	6 (50.0)	2 (16.7)	1 (8.3)	
	Others	3 (25.0)	8 (66.7)	1 (8.3)	0 (0.0)	
Marital status	Single	15 (6.2)	126 (51.6)	92 (37.7)	11 (4.5)	.012
	Married	6 (23.1)	14 (53.8)	5 (19.3)	1 (3.8)	
Average caring time (hours)	1–6	6 (20.0)	19 (63.3)	5 (16.7)	0 (0.0)	.001
	7–12	8 (12.5)	37 (57.8)	18 (28.1)	1 (1.6)	
	13–18	2 (4.6)	29 (67.5)	11 (25.6)	1 (2.3)	
	19–24	5 (3.8)	55 (41.3)	63 (47.4)	10 (7.5)	
Education	Primary	2 (2.6)	35 (45.4)	36 (46.7)	4 (5.3)	.040
	Secondary	2 (3.7)	32 (60.4)	16 (30.2)	3 (5.7)	
	High school diploma	9 (9.2)	51 (52.0)	35 (35.7)	3 (3.1)	
	Academic	8 (19.0)	22 (52.4)	10 (23.8)	2 (4.8)	
Job	Unemployed	12 (6.2)	94 (48.2)	78 (40.0)	11 (5.6)	0.052
	Employed	4 (18.2)	11 (50.0)	7 (31.8)	0 (0.0)	
	Self-employed	5 (9.5)	35 (66.0)	12 (22.6)	1 (1.9)	
Income	Insufficient for expenses	19 (7.4)	132 (51.4)	95 (37.0)	11 (4.2)	0.363
	Sufficient for expenses	2 (15.4)	8 (61.5)	2 (15.4)	1 (7.7)

**Table 3:** Factors associated with care burden by univariate liner regression (*N* = 270)

Variables	Subgroup	no (%)	B	SE	95% CI^*^	*P*-value
Age (year)	< 40	216 (80.0)	Ref.	–	–	–
	≥40	54 (20.0)	−4.15	2.27	−8.63, 0.33	0.069
Gender	Male	81 (30.0)	Ref.	–	–	–
	Female	189 (70.0)	5.80	1.97	1.92, 9.66	0.004
Relationship with patient	Parent	246 (91.2)	Ref.	–	–	–
	Sibling	12 (4.4)	−8.37	4.36	−16.97, 0.22	0.056
	Others	12 (4.4)	−12.46	4.36	−21.05, −3.87	0.005
Marital status	Married	244 (90.4)	Ref.	–	–	–
	Single	26 (9.6)	−9.05	3.05	−15.06, −3.04	0.003
Education	Primary	77 (28.5)	Ref.	–	–	–
	Secondary	53 (19.6)	−5.05	2.62	−10.20, 0.10	0.055
	High school diploma	98 (36.3)	−7.83	2.23	−12.23, −3.43	0.001
	Academic	42 (15.6)	−9.30	2.81	−14.84, −3.76	0.001
Income	Insufficient for expenses	257(95.2)	Ref.	–	–	–
	Sufficient for expenses	13(4.8)	−5.16	4.30	−13.55, 3.23	0.228
Job	Unemployed	195 (72.2)	Ref.	–	–	–
	Employed	22 (8.2)	−7.2	3.32	−13.74, −0.66	0.031
	Self-employed	53 (19.6)	−6.38	2.29	−10.89, − 1.88	0.006
Average caring time (hours)	1–6	30 (11.1)	Ref.	–	–	–
	7–12	64 (23.7)	6.11	3.12	−0.04, 12.26	0.051
	13–18	43 (15.9)	9.45	3.36	2.84, 16.06	0.005
	19–24	133 (49.3)	15.28	2.85	9.66, 20.90	< 0.001
Gender of child	Male	142 (52.6)	Ref.	–	–	–
	Female	128 (47.4)	−0.35	1.83	−3.96, 3.26	0.85
Age of child (year)	–	–	−0.93	0.22	−1.37, −0.50	< 0.001

^*^Confidence Interval

## Discussion

This cross-sectional study was conducted to determine the level of care burden and its related factors in the caregivers of children with cancer in Iran. In line with previous studies [4, 15, 17, 18, 28], most caregivers of children with cancer endured moderate to high care burden. Cancer diagnosis and treatment cause many psychological problems for the family of a child with cancer. Care burden is one of the most common psychological problems [29]. Multiple factors can cause care burden in the caregivers of children with cancer, including the challenges associated with cancer treatment, financial problems, lack of information about the course of the disease, feelings of inadequacy, worries about the future of the child, and disorganized family life [4, 15]. Caring for children with cancer is the main responsibility of the family, which puts a lot of care burden on the family members, especially parents. In this regard, the results of a study (2018) showed that the caregivers of patients with cancer were under a lot of stress and were always worried about an unpleasant event, thereby causing a lot of care burden [13].

Consistent with the study of Schrank et al. (2016) [20], the level of care burden was higher in the female caregivers than the male caregivers. Mother caregivers believe that no one can take care of a sick child as much as they do [30]. According to the Iranian culture, mothers play the main role of caregiver for the sick child. For this reason and considering the longer time that Iranian mothers spend with a sick child, it is not far-fetched to expect high care burden among them. However, the results of a study in Mexico (2019) showed that gender is not a predictor of care burden [31].

Single caregivers had less care burden than married caregivers. Contrary to the current study, the results of a study (2019) in Iran showed that married mothers had less care burden than single mothers [16]. Moreover, two studies in the Netherlands (2014) and Canada (2012) showed single mothers had high care burden [32, 33]. However, single caregivers seem to be more prone to financial problems and parenting stress than married caregivers [32, 33], which contributes to their high care burden. Differences in the results of these studies can be related to differences in the living conditions of caregivers and their individual characteristics.

Caregivers who were the first-degree relatives of the patient had more care burden than those who were the second-degree relatives. Evidence suggests that parents are the primary caregivers of children with chronic diseases that are present at all stages of diagnosis and treatment [6, 7, 14, 34]. Having a child with cancer can fundamentally change the family life, putting parents in a difficult and stressful situation and making them experience a lot of care burden.

In line with a previous study [14], the results showed that caregivers with high education level experienced less care burden than caregivers with low education level. However, some studies have indicated no relationship between education and care burden [16]. Those who are more educated may be better able to manage their stress and have more problem-solving skills [4, 16]. On the other hand, caregivers with low levels of education may have low socio-economic levels and therefore have less resources to meet the needs of patient care [19].

Consistent with previous studies [4, 18, 35], caregivers with good financial status had less care burden. This may be due to the fact that well-off families can receive more supportive services from the community or provide better treatment for the child, which can improve the patient’s clinical condition and reduce parental care burden [18]. In this regard, the results of a qualitative study (2021) in China showed that the cost of cancer treatment for families with sick children was high, which made them stressful. Furthermore, financial problems were one of the main reasons for stopping treatment in this study [35]. Inadequate income reduces the quality of life, which in turn increases perceived care pressure.

In line with previous studies [13, 18], the results showed that the amount of care burden increased significantly as the number of caring hours increased. Normally, when the hours of caring for a sick child are long, the caregivers expend more energy, which leads to an increase in perceived care burden.

In the present study, the amount of care burden decreased by an average of 0.9 units when the child’s age increased by one year. However, in some studies (2019), an inverse correlation has been reported between care pressure and child’s age [17, 18, 36]. Nevertheless, Arab et al. (2019) and Adib-Hajbaghery et al. (2019) reported the age of the child was not a predictor of caring stress [16]. It is expected that a younger sick child needs more care, which will naturally put a lot of care burden on caregivers.

In line with previous studies [14, 19] the employed caregivers had less care burden than the unemployed caregivers. However, some studies have shown no relationship between employment status and care burden [16]. Employed caregivers are usually financially independent and able to meet the different needs of the family and the sick child, but unemployed caregivers do not have a good financial situation, which reduces their quality of life and in turn leads to an increase in their caregiving pressure.

In the present study, no relationship was found between the sex of the sick child and care burden in the caregivers, which is in line with the results of the study of Motlagh et al. (2019) [17]. But in the study of Santo et al. (2009), this relationship was significant, and caregivers whose sick children were male had a greater care burden [30]. However, caring for a child with cancer, whether a boy or a girl, exposes their caregivers to a lot of care burden.

### Study limitations

The current study was conducted in a public hospital whose patients are usually from low-income groups in the community, thereby affecting the amount of care burden perceived by caregivers. The stage and degree of cancer in the children and the time from the diagnosis can affect the amount of care burden in caregivers, which was not considered in the current study due to the limited number of patients. The last limitation is related to the nature of cross-sectional studies. In these studies, it is not possible to determine the cause-and-effect relationships between the studied variables, and the current study is no exception to this limitation.

## Conclusion

In the current study, most caregivers of children with cancer experienced moderate to high care burden. Variables related to care burden included the caregiver’s gender, family relationship with the patient, marital status, education, occupation, length of caring hours, and age of the sick child. To reduce care burden, the health authorities need to take family-centered measures to reduce the caregiver burden. In this regard, referring low-income caregivers to support organizations, providing counseling services, as well as holding training workshops on caring for children with cancer can be useful. Future studies are suggested to investigate the effect of intervention measures on the care burden of caregivers of children with cancer.

## Data Availability

The identified datasets analyzed during the current study are available from the corresponding author on a reasonable request.

## Abbreviation

CBI: Caring Burden Inventory
